# Endoscopy- and Monitored Anesthesia Care-Assisted High-Resolution Impedance Manometry Improves Clinical Management

**DOI:** 10.1155/2018/9720243

**Published:** 2018-08-07

**Authors:** Kaci E. Christian, John D. Morris, Guofeng Xie

**Affiliations:** Division of Gastroenterology and Hepatology, University of Maryland School of Medicine, Veterans Affairs Maryland Health Care System, Baltimore, MD 21201, USA

## Abstract

**Background:**

High-resolution impedance manometry (HRiM) is the test of choice to diagnose esophageal motility disorders and is particularly useful for identifying achalasia subtypes, which often guide therapy. HRiM is typically performed without sedation in the office setting. However, a substantial number of patients fail this approach. We report our single-center experience on endoscopy-assisted HRiM under monitored anesthesia care (MAC) in adults to demonstrate the feasibility and effectiveness of this approach.

**Methods:**

Patients who had failed prior HRiM attempts received propofol under MAC. Patients then underwent an upper endoscopy, followed immediately by passage of a Diversateck HRiM motility catheter through the nares and under direct visualization into the stomach, often using the tip of the endoscope to guide the catheter. We then awakened the patients and asked them to perform 10 saline swallows.

**Results:**

We successfully completed HRiM studies in 14 consecutive patients. Six patients had achalasia; two had esophagogastric junction outflow obstruction; two had absent contractility; one had distal esophageal spasm; one had ineffective esophageal motility; and one had a normal study. The majority of these patients were treated successfully with targeted interventions, including per oral endoscopic myotomy, gastrostomy, botox injection, medical therapy, and dietary modifications.

## 1. Introduction

Esophageal manometry has become the gold-standard test to diagnose esophageal motility disorders and is also useful in the evaluation of gastroesophageal reflux disease (GERD), noncardiac chest pain, or systemic conditions that may lead to esophageal dysmotility. High-resolution impedance manometry (HRiM) with topography plotting incorporates impedance and manometry sensors, providing information on esophageal peristaltic patterns and pressures. Identification of specific esophageal motility disorders, especially subtypes of achalasia, is important, since this often guides therapeutic options [1]. The procedure is typically performed without sedation in the outpatient setting. However, some patients fail this approach due to a variety of reasons including poor tolerance or anatomic variants precluding intranasal intubation, coiling in the pharynx or esophagus, or hypersensitive gag reflex. In prior studies, 21% of high-resolution manometry studies were technically imperfect and 29% of those were imperfect due to inability to traverse the lower esophageal sphincter (LES) [2]. Twelve percent of the above-mentioned series of imperfect studies were due to inability to complete the minimum number of swallows for reasons including intolerance of the procedure [2], which include inability to intubate the nares or failure to traverse the LES. No standardized alternative techniques exist.

Previous reports trialed using through-the-scope manometric assessment revealed a good correlation between LES pressures obtained by standard manometry. However, these reports were limited by reduced peristaltic wave amplitudes due to the use of dry swallows [3]. Another group reported accurate diagnoses of achalasia and esophageal scleroderma by directly visualizing swallows during videoendoscopy [4]. However, according to the widely accepted Chicago classification, this technique lacks the metrics required for a diagnosis of a major motility disorder [5]. More recently, failure to perform transnasal manometry was circumvented by using an endoscopic-assisted over-the-wire technique, which utilized a water-perfusion motility catheter. Successful completion of the manometric study and diagnosis in this cohort resulted in treatment for achalasia (33.3%), change in medication (33.3%), and completion of preoperative assessment (27.7%) [6].

We report our single-center experience on endoscopy- and monitored anesthesia care- (MAC-) assisted high-resolution esophageal impedance manometry in adult patients to demonstrate the feasibility and effectiveness of this technique.

## 2. Materials and Methods

We evaluated patients who had failed prior attempts at manometry in the office setting for this study. All MAC- and EGD-assisted HRiM studies were performed within one week of prior failed attempts of unsedated HRiM tests. Subjects arrived at the endoscopy suite after fasting for a minimum of 6 hours. Topical anesthesia was provided with Cetacaine (benzocaine 14%, butamben 2%, and tetracaine hydrochloride 2%) spray to the throat and 5 mL of viscous lidocaine 2% solution to the nares. Patients were then sedated using propofol at the anesthetist's discretion in a monitored anesthesia care (MAC) setting. First, while, in the supine position, patients underwent a standard upper endoscopy using an Olympus GIF H180 or H190 gastroscope; an Olympus GIF XP190 scope was required in some cases. A Diversateck HRiM motility catheter was then passed through the right or left nostril into the esophagus and proximal stomach, under direct visualization and guidance with an endoscope. The tip of the catheter was centered directly above the esophagogastric junction (EGJ) prior to entering the stomach. One patient required insertion of a nasopharyngeal trumpeted airway to allow passage of the manometry catheter through the nares due to a history of prior craniofacial surgery. Once the manometry probe was in the correct position, the endoscope was withdrawn. To avoid damaging the motility catheter, endoscopic tools such as biopsy forceps or snares were not used. In some cases, simple endoscopic maneuvers such as opening the EGJ with the neonatal endoscope or gently nudging the tip of the catheter with an endoscope to redirect the motility probe were required to successfully place the motility catheter into the stomach. There was no visible damage to the motility catheter. After allowing sufficient time to awaken (usually 5 to 10 minutes), patients were given 10 consecutive 5-mL boluses of normal saline, followed by 5 mL of a viscous solution (Diversatek Healthcare Inc) as needed, while being in supine position for a total of 10 to 20 swallows. Esophageal muscle functions were recorded using the Diversatek ZVU software. The motility catheter was removed at the end of the procedure. On average, the EGD-assisted HRiM study took about 30 minutes versus 15 to 20 minutes for conventional HRiM test.

## 3. Results

As shown in Table 1, from January 1, 2017, to December 31, 2017, our institution performed high-resolution esophageal impedance manometry (HRiM) on 164 unique patients. Of these, 63 received manometry/impedance-pH tests to evaluate refractory GERD (38.4%); six manometry/impedance-pH tests were performed as part of lung transplant preoperative evaluation (3.7%); 91 manometry for evaluation of dysphagia (55.5%); and four manometry to evaluate atypical chest pain (2.4%). Forty-seven patients (29%) had an incomplete or limited study either at our institution or on previous attempts with outside providers. Of these, 14 patients were unable to tolerate the procedure due to excessive gagging or coughing (30%); in 20 patients, we were unable to pass the catheter through either nostril (43%), and, in 11 patients (23%), the catheter was unable to traverse the EGJ/LES or was coiled in the distal esophagus. We reviewed the charts of these patients, who either failed unsedated manometry or had known anatomic abnormalities limiting unsedated manometry, and found 14 consecutive patients who underwent MAC-assisted endoscopic probe placement. A brief clinical vignette is presented on these patients below (summarized and described in Tables 2 and 3). In summary, 11 of the 14 patients (78.6%) were diagnosed with a major motility disorder based on the most recent Chicago classification of esophageal disorders [7], eight of whom had either a subtype of achalasia or esophagogastric junction outlet obstruction (EGJOO). Five patients underwent procedural interventions including three peroral endoscopic myotomies (POEM) (21.4%), one botulinum toxin (Botox) injection (7.1%), and one percutaneous endoscopic gastrostomy (PEG) (7.1%). Three patients (21.4%) were treated with medical therapy, and, in the remaining six patients, we recommended dietary modification (28.6%) or continuation of previous therapy (14.3%).

**Patient 1** was a 32-year-old woman with a history of achalasia with a Heller myotomy at age of eight years. She developed recurrent dysphagia that had been either refractory or with only temporary response, to multiple pneumatic dilations at other institutions. She was unable to tolerate unsedated manometry. The manometry catheter was successfully placed at the time of sedated endoscopy with findings of patent esophagogastric junction (EGJ)/LES, severe ineffective motility and distal esophageal spasm, and a normal IRP consistent with her history of previously treated type III achalasia.

**Patient 2** was a 51-year-old woman with prior laparoscopic hiatal hernia repair and Toupet fundoplication with solid and liquid dysphagia and nausea who was being considered for surgical revision. Due to a history of craniofacial fractures and postsurgical anatomy and refusal to undergo unsedated manometry, she required direct visual guidance with an EGD scope for proper passage and positioning of the catheter; her manometry was normal.

**Patient 3** was a 62-year-old woman with a prior Nissen fundoplication for GERD followed by repair of type III paraesophageal hernia with Belsey-Mark IV fundoplication two years later who developed dysphagia and regurgitation. She was unable to tolerate unsedated manometry due to severe gagging. Subsequently, the motility catheter was successfully advanced into the proximal stomach with MAC-assisted endoscopic guidance and showed findings of severe ineffective esophageal motility.

**Patient 4 **was a 71-year-old woman with reflux symptoms and pulmonary fibrosis undergoing evaluation for lung transplantation. Unsedated manometry was attempted but unsuccessful due to inability to traverse the EGJ with the motility catheter. Under endoscopic guidance, the catheter was visualized abutting the distal esophagus just above the EGJ and was successfully guided into the proximal stomach with the assistance of a neonatal endoscope (Olympus GIF XP190). Her motility findings were consistent with type II achalasia. After discussing risks and benefits with the transplant team, patient deferred Heller myotomy and transplant listing.

**Patient 5** was a 52-year-old woman with a six-month history of progressively worsening solid and liquid dysphagia associated with a >50 lbs weight loss. Patient discomfort and inability to traverse the EGJ precluded her from completing unsedated manometry. Via endoscopy-assisted manometry, a diagnosis of type II achalasia was made and the patient proceeded to POEM with improvement in Eckardt score from 10 (preop) to 3 (postop).

**Patient 6** was an 18-year-old man with progressive dysphagia and an associated 65-lb weight loss who was unable to tolerate insertion of the manometry probe due to severe gagging and discomfort. He underwent MAC-assisted probe placement; direct visualization was needed due to a dilated esophagus and tight gastroesophageal junction. He was diagnosed with type II achalasia and proceeded to POEM with improvement in Eckardt score from 9 to 0.

**Patient 7** was an 85-year-old man with solid and liquid dysphagia and an inability to traverse the gastroesophageal junction during prior attempts at manometry. He had undergone prior endoscopies with balloon dilation of the mid esophagus with minimal relief. Barium esophagram revealed a corkscrew appearance. He underwent endoscopy-assisted manometry that showed distal esophageal spasm; he was referred for Botox injection 5 cm above the EGJ with significant symptomatic improvement.

**Patient 8** was a 63-year-old man with a history of a bilateral lung transplant due to silicosis with episodes of recurrent aspiration and a barium esophagram concerning for a gastroesophageal junction stricture. Due to this known abnormality, direct visualization following standard upper endoscopy exam was used to help advance the manometry probe past a tight lower esophageal sphincter. A diagnosis of EGJ outflow obstruction was made; due to fragile pulmonary status following frequent aspiration events, a percutaneous endoscopic gastrostomy tube was placed following this diagnosis.

**Patient 9** was a 65-year-old man with a history of dysphagia who failed awake manometry due to inability to pass the probe through his nares with looping in the posterior oropharynx. He had a history of prior craniofacial surgery. After MAC sedation, attempt to insert the motility catheter via either nostril failed. A trumpeted nasal airway was used to allow passage of the probe through his nares and into the esophagus. Due to difficulty traversing the EGJ, an Olympus GIF XP190 scope was used to help guide the probe into proximal stomach. Manometry revealed EGJ outflow obstruction and he was treated successfully with a calcium channel blocker.

**Patient 10** was a 66-year-old man with two years of worsening dysphagia and 40-lb weight loss referred from another institution after an EGD showed a dilated esophagus and inability to traverse the EGJ with the endoscope, suggestive of achalasia. He underwent repeat EGD at our institution, noting a tight EGJ/LES that was traversed with moderate pressure. Under direct visualization, the manometry catheter was placed into the distal esophagus but was unable to be advanced further due to patient's oxygen desaturation that resolved after propofol infusion was discontinued. Manometry revealed aperistalsis with panesophageal pressurization that, in conjunction with the EGD results and barium esophagram showing tapering of the distal esophagus, was strongly suggestive of type II achalasia. The patient was referred for and underwent successful POEM.

**Patient 11** was a 58-year-old man with a history of bilateral lung transplant due to cryptogenic organizing pneumonia referred for recurrent aspiration pneumonia and abnormal esophagram with poor antegrade peristalsis and distal intraesophageal reflux. After failing unsedated probe placement, he underwent EGD with endoscopic placement of the motility catheter into the proximal stomach. Manometry revealed ineffective esophageal motility.

**Patient 12** was a 24-year-old woman with a history of Type I achalasia who had undergone Heller myotomy seven years earlier and complained of progressively worsening dysphagia. Unsedated manometry was unsuccessful, as the probe was not able to enter the esophagus. During sedated probe placement with direct visualization, the probe was not able to enter esophagus via the right pyriform sinus. The probe was repositioned to the left pyriform sinus and able to be advanced into proximal stomach. EGD showed evidence of prior myotomy and patent EGJ/LES. Postsurgical type I achalasia was diagnosed based on manometry findings and dietary modifications were recommended.

**Patient 13** was a 61-year-old man with a two-year history of solid and liquid dysphagia. An esophagram revealed smooth narrowing of the gastroesophageal junction with incomplete relaxation, a bird's beak appearance, and esophageal dysmotility suggestive of achalasia. Unsedated manometry was attempted and revealed absent peristalsis. However, the probe had looped in the distal esophagus and did not traverse the EGJ. Due to the uncertainty in diagnosis, the patient underwent endoscopy-guided placement of the motility catheter into the proximal stomach under MAC. EGD showed patent EGJ/LES. Manometry confirmed absent contractility and dietary modifications were recommended.

**Patient 14** was a 75-year-old woman with a history of atypical chest pain and dysphagia who did not tolerate unsedated manometry due to gagging. She underwent successful placement of the manometry catheter into the proximal stomach with EGD guidance under MAC. EGD showed patent EGJ/LES. Manometry findings were consistent with absent contractility and she was currently treated with Bethanechol.

## 4. Discussion

We report our experience with MAC/endoscopy-assisted HRiM and clinical outcomes in 14 patients in whom unsedated manometry had previously failed or was contraindicated. The approach was used in a subset of our patients in whom manometric data was required prior to referral for possible invasive procedures or in whom there was diagnostic uncertainty. Notably, all procedures were successful without any peri- or postprocedural complications.

**Figure 1:**
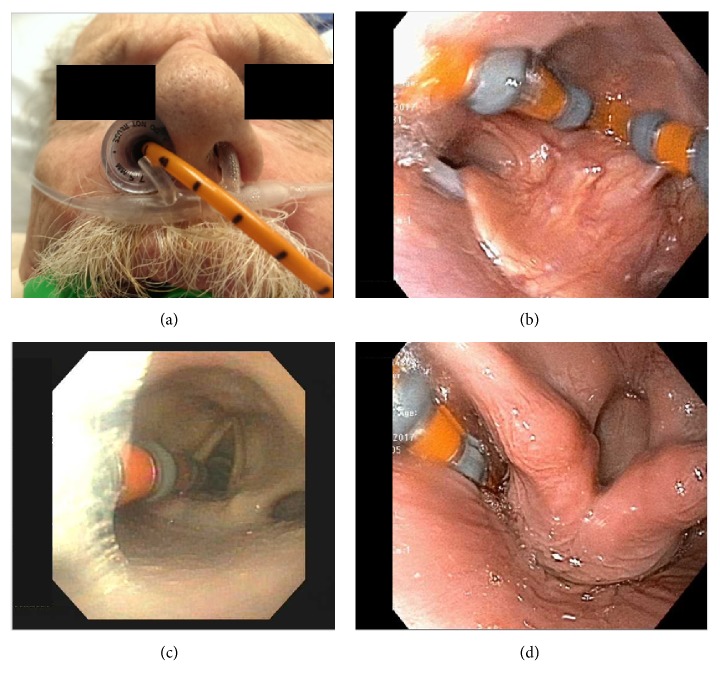
Examples of nasopharyngeal and laryngeal issues addressed with MAC-assisted endoscopic placement. (a) Nasal trumpet used for deviated septum and prior sinus surgery. (b) Coiling of the motility catheter in the posterior oropharynx at the vallecula of the epiglottis. (c) Motility catheter visualized in the trachea prior to being endoscopically guided through the upper esophageal sphincter (UES). (d) Successful placement of the motility catheter through the UES.

**Figure 2:**
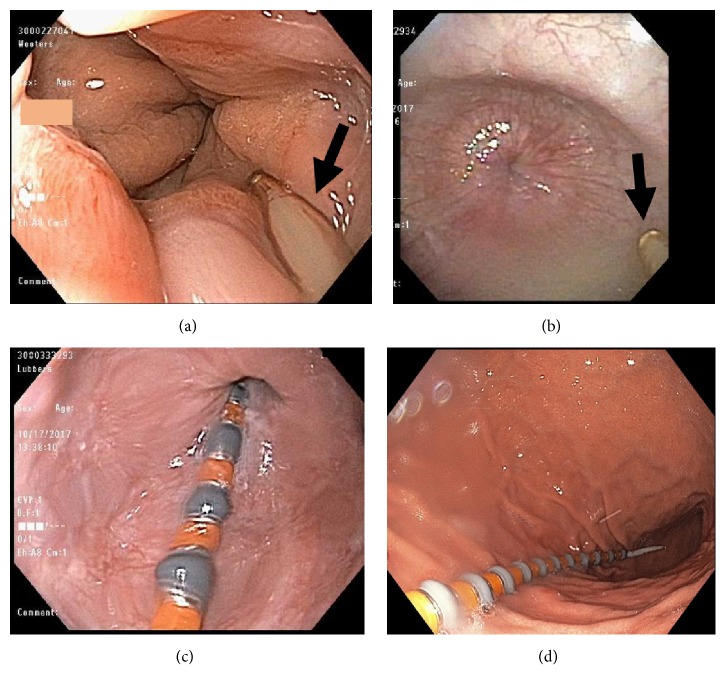
Examples of esophagogastric junction (EGJ) issues addressed with MAC-assisted endoscopic placement. (a) Motility catheter (arrow) hung up at hiatal hernia. (b) Motility catheter (arrow) impeded at tight lower esophageal sphincter (LES) in a patient with achalasia with a tight LES. (c) Motility catheter (arrow) visualized passing through the EGJ under direct visualization. (d) Endoscopic confirmation of successful placement of the motility catheter through the LES into the stomach.

This technique effectively overcame anatomical issues related to esophageal tortuosity, distal esophageal dilation, tight LES, deviated septum, or prior craniofacial surgery, respectively. Additionally, patient-related issues such as excessive coughing or gagging and inability to complete the study due to excessive discomfort were managed. Lastly, direct visualization was able to resolve uncertainty in cases with questionable prior manometric findings related to uncertain probe placement. Examples of nasopharyngeal/laryngeal and esophageal/EGJ issues of manometry probe placement are shown in Figures 1 and 2, respectively.

In our experience, the use of endoscopy-assisted HRiM allowed for the successful diagnosis of dysphagia and subsequent treatment with a procedural intervention in a substantial portion of our cohort (35.7% - POEM 21.4%, Botox injection 7.1%, PEG 7.1%), as well as medical therapy in three patients (21.4%), or dietary modifications in four (28.6%). In addition, in four patients (28.6%), the manometry findings supported the decision to defer further interventional procedures, including two patients with previously treated achalasia found to have a normal IRP, and two patients with prior hiatal hernia repair under consideration for surgical revision. Lastly, in one pre-lung transplant patient (Patient #4), a new diagnosis of achalasia assisted the patient in deciding not to pursue lung transplantation after a risk-benefit discussion with the transplant team.

Limitations of this study include the small sample size, retrospective review of the cases, and lack of a controlled comparative group. However, our study does show the potential benefit of pursuing objective HRiM data and a definitive diagnosis in patients who fail initial attempts at unsedated manometry. Due to the additional expense and small increase in risk of complication due to the endoscopic procedure and required sedation, we only selected a subset of our patients in whom there was a particularly high suspicion of a major motility disorder in which a procedural intervention might be beneficial. Also, we selected patients in whom the decision to recommend a surgical procedure was, in large part, dependent on the manometry findings. Furthermore, obtaining a definitive manometric diagnosis for spastic esophageal disorders as the cause of dysphagia becomes increasingly important as POEM emerged as an attractive treatment option [8, 9].

Another potential limitation of this technique is that the effect of anesthesia on esophageal motility is uncertain. Topical anesthetics do not influence the pharyngeal phase of swallowing, once the swallowing reflex is triggered [10]. Propofol does not alter the gastroesophageal pressure gradient but minimally decreases lower esophageal sphincter pressure at higher doses, an effect not seen with moderate doses [11]. Propofol, which is quickly redistributed from the central nervous system to peripheral tissues, has short duration of action [12]. This short duration of action and quick recovery have allowed for the frequent use of propofol in endoscopy to facilitate patient throughput [13]. In our cohort of patients, this feature also allowed a necessary short turnaround time to obtain manometric data with awake supine swallows. Indeed, the large proportion of achalasia and other major motility disorder diagnoses in our patients provides additional evidence that the residual effects of propofol are unlikely to confound acquisition of manometric data when performed after a short wash out period.

In conclusion, endoscopy- and MAC-assisted HRiM can ensure completion of esophageal motility studies in patients otherwise unable to achieve definitive manometric diagnoses. In selected patients with suspected major motility disorders such as achalasia, EGJOO, or spastic esophageal motility disorders, our approach can help guide therapy and result in successful treatment outcomes.

## Figures and Tables

**Table 1:** Indication for manometry testing with failure rate as well as reason for failed study.

Indication	N	Failed or limited study	% failed
Refractory GERD	63	21	33%

Lung transplant evaluation	6	1	17%

Dysphagia	91	25	27%

Chest pain	4	0	0%

TOTAL	164	47	29%

Reason for failure	N	% of total	

Gagging	14	29.8%	

Nostrils	20	42.6%	

LES or esophagus	11	23.4%	

Unclear	2	4.3%	

TOTAL	47	100.0%	

**Table 2:** Description and outcomes of MAC- and endoscopy-assisted manometry cases.

			N	%
Age			14	

Sex				

	Women		7	50%

	Men		7	50%

Indication				

	Dysphagia		8	57.1%

	Recurrent dysphagia		2	14.3%

	Recurrent aspiration, dysphagia		2	14.3%

	Lung transplant evaluation, GERD		1	7.1%

	Atypical chest pain		1	7.1%

Reason for requiring endoscopic probe placement				

	Inability to traverse LES		5	35.7%

	Gagging		3	21.4%

	Patient discomfort		2	14.3%

	EGD indicated		2	14.3%

	History of craniofacial fractures		1	7.1%

	Looping posterior oropharynx		1	7.1%

Findings/Diagnosis				

	Major motility abnormality		11	78.6%

		*Type II achalasia*	4	28.6%

		*Previously treated achalasia*	2	14.3%

		*EGJOO*	2	14.3%

		*Absent contractility*	2	14.3%

		*DES*	1	7.1%

	IEM		2	14.3%

	Normal		1	7.1%

Treatment recommendations				

	Interventions		5	35.7%

		*POEM*	3	21.4%

		*Botox injection*	1	7.1%

		*PEG*	1	7.1%

	Medical therapy		3	21.4%

	Dietary modification		4	28.6%

	Other		2	14.3%

**Table 3:** Summary of MAC- and endoscopy-assisted manometry cases.

*#*	*Age*	*Sex*	*Pertinent history*	*Indication for EGD-Assistance*	*Diagnosis*	*Recommendation*	*Outcome*
1	32	F	Achalasia s/p HM	Patient discomfort	Type III achalasia s/p HM	Diet modification	Not available

2	51	F	HH repair, Toupet fundoplication	Prior craniofacial fractures	Normal	N/A	Stable symptoms

3	62	F	Type III PEH s/p fundoplication	Severe gagging	Severe IEM s/p fundoplication	Diet modification	Improved dysphagia

4	71	F	Pulmonary fibrosis, GERD	Inability to traverse LES	Type II achalasia	Follow up with pulmonary	Deferred HM, transplant listing

5	52	F	Progressive dysphagia w/ weight loss	Inability to traverse LES	Type II achalasia	POEM performed	Improved dysphagia; weight gain

6	18	M	Dysphagia w/ weight loss	Severe gagging	Type II achalasia	POEM performed	Improved dysphagia; weight gain

7	85	M	Corkscrew esophagram	Inability to traverse LES	DES	Botox injection performed	Improvement in dysphagia

8	63	M	S/p lung transplant, abnormal esophagram	EGD for possible GEJ stricture	EGJOO	PEG for enteral nutrition	Tolerated PEG; stable lung symptoms

9	65	M	Prior craniofacial surgery	Oropharyngeal looping	EGJOO	Calcium channel blocker	No follow up available

10	66	M	Dysphagia w/ weight loss	Inability to traverse LES	Type II achalasia	POEM performed	Improved dysphagia; weight gain

11	58	M	S/p lung transplant, abnormal esophagram	No prior EGD	IEM	GERD management	Stable

12	24	F	Type I achalasia s/p HM	Patient discomfort	Type I achalasia s/p treatment	Diet modification	Not available

13	61	M	Bird's beak esophagram	Probe looping	Absent contractility	Dietary modification	Long hospital stay

14	75	F	GERD, prior candida esophagitis	Gagging	Absent contractility	Bethanechol	Not available

HH: hiatal hernia; HM: Heller myotomy; PEH: paraesophageal hernia; LES: lower esophageal sphincter; GEJ: gastroesophageal junction; IEM: ineffective esophageal motility; DES: diffuse esophageal spasm; EGJOO: esophagogastric junction outflow obstruction; POEM: peroral endoscopic myotomy.
